# FOXP3^+^ Lymphocyte Density in Pancreatic Cancer Correlates with Lymph Node Metastasis

**DOI:** 10.1371/journal.pone.0106741

**Published:** 2014-09-05

**Authors:** Yongjian Jiang, Zunguo Du, Feng Yang, Yang Di, Ji Li, Zhongwen Zhou, Venu G. Pillarisetty, Deliang Fu

**Affiliations:** 1 Department of Pancreatic Surgery, Huashan Hospital, Fudan University, Shanghai, China; 2 Department of Pathology, Huashan Hospital, Fudan University, Shanghai, China; 3 Department of Surgery, University of Washington, Seattle, Washington, United States of America; Centro di Riferimento Oncologico, IRCCS National Cancer Institute, Italy

## Abstract

**Objective:**

To determine if the density of FOXP3^+^ lymphocytes in primary tumors and lymph nodes in pancreatic cancer correlates with the presence of lymph node metastases.

**Methods:**

FOXP3^+^ lymphocyte density in primary pancreatic cancer tissue and draining lymph nodes was measured using immunohistochemistry. We analyzed the clinical and pathological aspects associated with the accumulation of FOXP3^+^ lymphocytes in pancreatic cancer. We also analyzed the correlation of density of FOXP3^+^ lymphocytes in lymph nodes with the nodal status and distance from the primary tumor.

**Results:**

FOXP3^+^ lymphocyte density in pancreatic cancer was significantly higher than in paratumoral pancreatic tissue. The density of FOXP3^+^ lymphocytes in local tumor tissue correlated significantly with the histological grade and overall lymph node status. Furthermore, FOXP3^+^ lymphocyte density was significantly higher in positive lymph nodes than in negative ones, while it had no correlation with the distance of the lymph node from the primary tumor.

**Conclusion:**

FOXP3^+^ lymphocyte density in primary tumor tissue in patients with pancreatic cancer correlates with lymph node metastasis. Lymph nodes containing metastases having higher FOXP3^+^ lymphocyte densities than do negative lymph nodes.

## Introduction

Pancreatic ductal adenocarcinoma, commonly known as pancreatic cancer, is amongst the most aggressive of human malignancies. Radical surgical resection of localized disease provides a small chance of cure; however, it is rarely indicated because major vascular invasion and/or distant metastases are frequently discovered at diagnosis. Even when surgical resection is possible, metastasis and recurrence are especially prominent in pancreatic cancer [1].

Much of the traditional focus of cancer research has involved studying the genotypic and phenotypic changes that underlie cancer development, progression, and metastasis. In contrast to these cell-autonomous aspects of solid tumors, current studies are shedding light on other critical elements of the tumor microenvironment [2]. Based upon such studies, it is becoming clear that immune cells and their associated cytokines are important components of the tumor microenvironment of pancreatic cancer [3]. Regulatory T cells (Treg), which were initially defined in the context of autoimmune diseases and are known to suppress T cell-mediated immune responses, are now believed to play a crucial role in impeding immune surveillance against cancer and hampering the development of effective antitumor immunity. It was reported that Treg increased in the peripheral blood, tumor microenvironment and ascites in variety of malignancies and appear to interfere with multiple aspects of anti-tumor immunity [4]–[7].

In pancreatic cancer patients, it has been well documented that the prevalence of Treg both in peripheral blood and tumor microenvironments is elevated, and that Treg infiltration correlates directly with tumor pathological stage and prognosis [8]–[12]. We recently confirmed that pancreatic cancer is infiltrated by FOXP3^+^ putative Treg, and this served as a potential explanation for the poor survival of patients despite the high prevalence of CD8^+^ T cells [13]. In that study, we also used flow cytometry to confirm that the FOXP3^+^ cells were also CD3^+^CD4^+^CD25^+^CD127^−^, thus strengthening the suggestion that FOXP3 can serve as a useful marker for Treg in pancreatic cancer.

The presence or absence of lymph node metastases is the strongest predictor of outcomes following surgical resection of pancreatic cancer; however, little is known about the relationship between Treg infiltration of pancreatic cancer and lymph node metastasis. Therefore, we tested the hypothesis that elevated levels of FOXP3 immunostaining in primary tumors and regional lymph nodes would predict the presence of lymph node metastases in pancreatic cancer.

## Patients and Methods

### Patients

In this study, we collected the clinical and pathological data of 50 patients who underwent radical resection for ductal adenocarcinoma of the pancreatic head between April 2005 and December 2008 at Huashan Hospital, Fudan University. Patients with a history of autoimmune disease and chronic/acute infection, and those who had received radiotherapy or chemotherapy before their operation were excluded. The nomenclature of the retrieved lymph nodes and the pathological staging was based on the *Second English Edition of the Classification of Pancreatic Carcinoma* proposed by the Japanese Pancreatic Society (JPS) [14]. The draining lymph nodes from the first, second and third groups are referred to N1, N2 and N3, respectively. This study was approved by the Ethics Committee of Huashan Hospital, Fudan University, Shanghai, China (NO. 2012215). Patient records were anonymized and de-identified prior to analysis.

### Immunohistochemistry

We reviewed the hematoxylin-eosin-stained sections of pancreatic tumor and paratumoral (adjacent non-neoplastic pancreatic parenchyma) tissues, and sampled lymph nodes, from patients with histologically confirmed pancreatic ductal adenocarcinoma. The corresponding formalin-fixed, paraffin-embedded tissues were collected, and 5-µm thick sections were prepared. Immunohistochemical staining of FOXP3 was performed using the avidin–biotin complex horseradish-peroxidase method. Briefly, paraffin sections were first deparaffinized and hydrated, and then subjected to a heat-induced antigen retrieval step (EDTA at pH 7.8). Endogenous peroxidase activity was blocked, and the slides incubated in 0.3% H_2_O_2_. The nonspecific binding sites were blocked with Protein Block (RE7102; Novocastra). The slides were then incubated with primary monoclonal antibodies against the FOXP3 protein (clone ab10563, 1∶50; Abcam). The bound secondary antibodies were visualized with diaminobenzidine (Dako) and counterstained with Harris hematoxylin. Negative control staining was performed with the primary antibody omitted.

### FOXP3^+^ lymphocyte enumeration

Each section was evaluated independently by two pathologists (Du ZG, Zhou ZW) who were blinded to the patients' clinical and pathological profiles. Cells positive for FOXP3 were counted in five randomly selected areas under high magnification (×400) (Olympus, Japan) and expressed as the average number of positive cells per high-power field (/HP). Discrepancies between the two pathologists were reviewed jointly to arrive at a consensus.

### Data analysis

Continuous variables are expressed as mean ± standard deviation (SD), and categorical variables are expressed as frequencies (%). Pearson χ^2^ and multiple logistic regressions were used for the univariate and multivariate analysis. Overall survival was estimated using the Kaplan-Meier method. The Log-rank test and Cox proportional hazards models were applied for univariate and multivariate analyses of risk factors for survival, respectively. All p values and 95% confidence intervals [CI] were estimated in a two-tailed fashion. Differences were considered statistically significant at P<0.05. Data were analyzed using SAS version 9.3 (SAS Institute Inc., Cary, NC, USA).

## Results

### General data

We identified 50 patients (29 males and 21 females; median age: 61 years; range: 20–79 yrs.) with adenocarcinoma of the pancreatic head who underwent pancreaticoduodenectomy (n = 48) or total pancreatectomy (n = 2). In addition to primary tumors, we analyzed a total of 157 lymph nodes, with 70 lymph nodes from N1 (including 24 positive and 46 negative lymph nodes from the posterior and anterior aspects of the head of the pancreas), 46 lymph nodes from N2 (including 11 positive and 35 negative lymph nodes from the areas surrounding the superior mesenteric artery or the hepatoduodenal ligament), and 41 lymph nodes from N3 (including 3 positive and 38 negative lymph nodes from the area surrounding the abdominal aorta).

### Density of FOXP3^+^ lymphocytes in tumor and paratumoral pancreatic tissue

**Figure 1:**
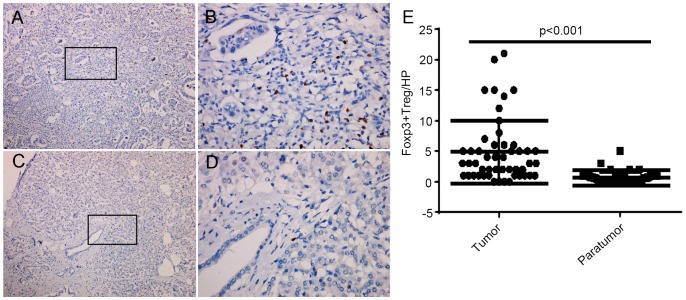
Comparison of FOXP3^+^ lymphocyte infiltration between pancreatic cancer and paratumoral tissue. Immunohistochemical staining of FOXP3^+^ lymphocytes in pancreatic cancer (A: low magnification, ×100; B: high magnification, ×400) and paratumoral pancreatic tissue (C: low magnification, ×100; D: high magnification, ×400): The FOXP3^+^ lymphocytes were defined as the cells with brown-staining nuclei in mononuclear lymphocytes. (E) Differences in FOXP3^+^ lymphocyte density between pancreatic cancer tissue and paratumoral pancreatic tissue. (p<0.01).

Consistent with prior reports, FOXP3^+^ lymphocytes infiltrated the primary tumors, and were found scattered throughout the interstitial regions of the tumor (**Figure 1A & B**). Few FOXP3^+^ lymphocytes were observed infiltrating the paratumoral pancreatic tissue (**Figure 1C & D**). The density of FOXP3^+^ lymphocytes in pancreatic cancer was significantly higher than that in paratumoral pancreatic tissue (4.8±5.2/HP vs. 0.47±0.94/HP, p<0.001, **Figure 1E**).

FOXP3^+^ lymphocyte density did not correlate with age, gender or T stage (including the tumor size and the local infiltration) of pancreatic cancer (**Table 1**). The density of FOXP3^+^ lymphocytes was higher in tumors with higher histological grade (well differentiated: 1.3±1.0/HP; vs. moderately differentiated: 4.3±4.5/HP vs. poorly/undifferentiated: 7.9±7.8/HP, p = 0.014). The density of FOXP3^+^ lymphocytes was significantly higher in pancreatic cancers with lymph node metastasis compared to those without lymph node metastasis (6.7±5.9/HP vs. 2.3±2.6/HP, p = 0.002). Similarly, there was a trend towards higher density of FOXP3^+^ lymphocytes in advanced-stage pancreatic cancer than early-stage disease. (I, II: 2.7±3.1 vs. III, IV: 5.5±5.6, p = 0.094).

**Table 1:** Density of FOXP3^+^ lymphocytes in pancreatic tumor tissue.

Variables	No. of patients	Density of FOXP3^+^ lymphocytes in tumor (/HP)	*P* value
**Age**			0.11
** ≤61**	25	3.6±3.3	
** >61**	25	6.0±6.4	
**Gender**			0.27
** Male**	29	5.5±6.0	
** Female**	21	3.8±3.6	
**Tumor stage**			0.59
** T1**	10	3.3±3.3	
** T2**	13	6.0±6.7	
** T3**	24	4.6±4.8	
** T4**	3	6.8±6.6	
**Histologic grade**			0.01
** Well differentiated**	7	1.3±1.0	
** Moderately differentiated**	30	4.3±3.5	
** Poorly/undifferentiated**	13	7.9±7.8	
**Lymph node status**			<0.01
** Node negative**	22	2.3±2.5	
** Node positive**	28	6.7±5.9	
**Pathological stage**			0.09
** I+II**	13	2.7±3.1	
** III+IV**	37	5.5±5.6	

### Density of FOXP3^+^ lymphocytes in draining lymph nodes

**Figure 2:**
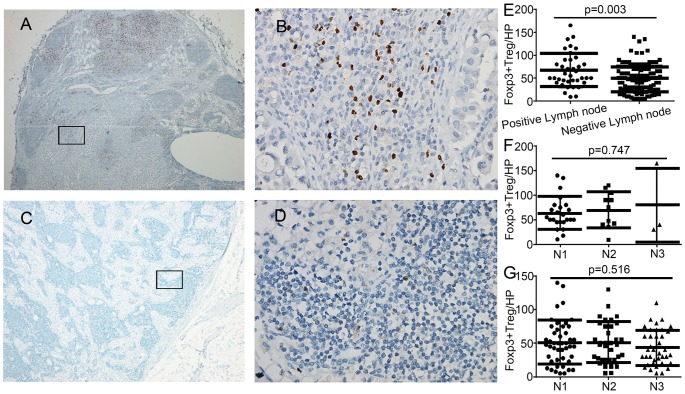
FOXP3^+^ lymphocytes in the draining lymph nodes of pancreatic cancer patients. Immunohistochemical staining of FOXP3^+^ lymphocytes in a positive lymph node (A: low magnification, ×40; B, high magnification, ×400) and a negative lymph node(C: low magnification, ×40; D: high magnification, ×400), and a comparison of the FOXP3^+^ lymphocyte densities in positive and negative lymph nodes (E) (p = 0.03), among different groups in positive lymph nodes (F) (p = 0.75), and among different groups in negative lymph nodes (G) (p = 0.52).

FOXP3^+^ lymphocytes accumulated in positive lymph nodes, primarily around the metastatic foci, at a density significantly higher than was observed in the negative lymph nodes (66.2±36.9/HP vs. 48.1±29.9/HP, p = 0.003, **Figure 2A–E**). No significant difference in density of FOXP3^+^ lymphocytes was observed in the different lymph nodes groups for positive (N1 vs. N2 vs. N3: 63.0±2.7/HP vs. 69.9±37.0/HP vs. 78.3±75.2/HP, p = 0.747, **Figure 2F**) or negative lymph nodes (N1 vs. N2 vs. N3: 50.4±32.5/HP vs. 50.0±30.3/HP vs. 43.4±26.4/HP, p = 0.516, **Figure 2G**).

### FOXP3^+^ lymphocyte infiltration of pancreatic cancer tumor is an independent risk factor for lymph node metastasis

Lymph node metastasis is the most important prognostic indicators of resectable pancreatic cancer. The median density of FOXP3^+^ lymphocytes (4/HP) in each tumor was considered to be the cutoff level and was analyzed in comparison to the patients' age, gender, histological grade and T stage of the tumor to identify the risk factors of lymph node metastasis in pancreatic cancer. Univariate and multivariate analyses confirmed that high intratumoral density of FOXP3^+^ lymphocytes is an independent risk factor for nodal metastases (**Table 2**).

**Table 2:** Univariate and multivariate analysis of relevant factors correlated with lymph node metastases of pancreatic cancer.

Univariate analysis				Multivariate analysis			
Variables	*n*	Number of node-positive patients	Metastatic rate	*P* value	Odds ratio	95% confidence interval	*P* value
**Age**				0.57	1.00	0.16–2.12	0.93
** ≤61**	25	13	52.0%				
** >61**	25	15	60.0%				
**Gender**				0.31	0.58	0.16–2.12	0.41
** Male**	29	18	62.1%				
** Female**	21	10	47.6%				
**Tumor stage**				0.71	1.27	0.58–2.80	0.55
** T1**	10	4	40.0%				
** T2**	13	8	61.5%				
** T3**	24	14	58.3%				
** T4**	3	2	66.7%				
**Histological grade**				0.06	1.29	0.42–3.96	0.66
** Well-differentiated**	7	1	14.3%				
** Moderately- differentiated**	30	19	63.3%				
** Poorly/undifferentiated**	13	8	61.5%				
**Density of Foxp3^+^ lymphocytes in tumor**				<0.01	1.36	1.05–1.77	0.02
** ≤4/HP**	30	12	40%				
** >4/HP**	20	16	80%				

### The intratumoral density of FOXP3^+^ lymphocytes tended to correlate with prognosis following radical resection

**Figure 3:**
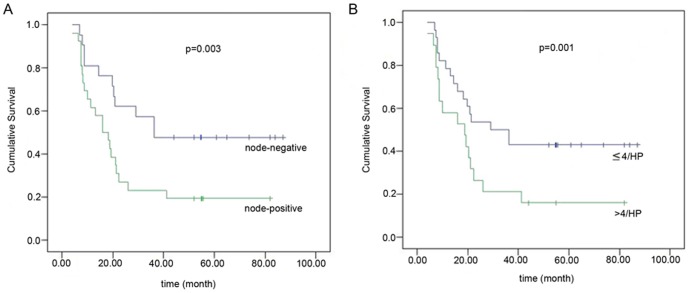
Cumulative survival analysis in 47 pancreatic head cancer patients after radical resection. Lymph node status (p = 0.03) (A) and density of FOXP3^+^ lymphocytes in pancreatic cancer tissue (p = 0.01) (B) were significantly correlated with the prognosis of pancreatic head cancer after radical resection.

Of the 50 patients in our study who underwent radical resection, we obtained the survival status and survival time for 47 patients through outpatient clinic or telephone follow-up. The median follow-up time was 21 months (4–87 months). In the univariate analysis, both the lymph node status and the density of FOXP3^+^ lymphocytes in the tumors correlated with the prognosis of the pancreatic cancer patients following radical resection (as shown in **Figure 3** and **Table 3**). A multivariate analysis incorporating the two factors suggested that the FOXP3^+^ lymphocyte density in tumor holds promise as an independent prognostic factor, however this did not reach statistical significance (p = 0.06).

**Table 3:** Univariate and multivariate analyses of prognostic factors in pancreatic cancer after radical resection.

Univariate analysis			Multivariate analysis	
Variables	HR(95% confidence interval)	*P* value	HR(95% confidence interval)	*P* value
**Age**	1.71(0.85–3.45)	0.13	-	-
**Gender**	1.01(0.50–2.05)	0.97	-	-
**Tumor stage**	1.47(0.96–2.24)	0.08	-	-
**Histological grade**	1.71(0.97–3.00)	0.06	-	-
**Lymph node status**	2.3(1.10–4.80)	0.03	1.88(0.86–4.11)	0.11
**Pathological stage**	2.09(0.86–5.08)	0.11	-	-
**Density of Foxp3^+^ lymphocyte in tumor**	1.09(1.02–1.15)	0.01	1.07(1.00–1.14)	0.06

## Discussion

In this study, we evaluated the distribution of FOXP3^+^ putative Treg in pancreatic cancer primary tumors and draining lymph nodes, and determined its correlation with critical clinicopathological features. As FOXP3 is the most widely accepted single immunohistochemical marker for Treg and has been shown to have some prognostic value, we confirmed that FOXP3 accumulated in the nuclei of small, round mononuclear lymphocytes that were clearly distinct from tumor cells [10]–[13]. Importantly, our study demonstrated a new association between the density of FOXP3^+^ lymphocytes in pancreatic cancer and lymph node metastasis. Furthermore, we have made the novel observation that FOXP3 expression is higher in lymph nodes containing pancreatic cancer metastases than in those that are negative for carcinoma.

Consistent with prior studies [10]–[13], we found that the density of FOXP3^+^ lymphocytes in pancreatic cancer correlated significantly with the histologic grade of the tumor, regardless of the patients' age, gender, tumor size, or local invasion. Differences in cell phenotype, chemokine expression, metabolism, oxidative status, and pH of the tumor microenvironment among different histological grades of pancreatic cancer may affect FOXP3^+^ lymphocyte density in tumor tissues; however, the precise mechanisms driving Treg infiltration of human pancreatic cancer has not yet been elucidated.

Lymph node metastasis is clearly an important event in solid tumors pathogenesis, especially pancreatic cancer [15]–[18]. As lymph nodes are the sites of antigen presentation and immune response initiation, they may serve as early barriers that prevent tumor cell entry into the systemic circulation [19]. The local microenvironment of lymph nodes is vital in setting the course of subsequent immune responses. However, prior studies have not demonstrated a relationship between FOXP3 infiltration and nodal status. Hiraoka et al. [10] had previously studied both primary pancreatic cancer tumors and lymph nodes, however the discrepancy between our finding and theirs may be due to our enumeration of total FOXP3^+^ cells rather than the ratio of FOXP3^+^ to CD4^+^ cells. We hypothesized that the absolute number of FOXP3^+^ putative Treg may, in fact, be more relevant in determining effects on the evolving anti-tumor immune response.

In this study, we analyzed 157 lymph nodes from the 50 patients, and found significantly higher density of FOXP3^+^ lymphocytes in positive lymph nodes compared with negative ones. While levels of FOXP3^+^ lymphocytes in lymph nodes correlated with the presence or absence of metastases, this was not independently affected by the distance of the lymph nodes from the primary pancreatic cancer. Deng et al. [20] found similar results in colorectal cancer, however this has not been previously shown in pancreatic cancer. There are essentially two mechanisms to explain our findings. First, immune tolerance mediated by FOXP3^+^ lymphocytes may contribute to the survival and proliferation of tumor cells that drain to the lymph nodes and ultimately form foci that are visible under light microscopy. Alternatively, the presence of tumor cells in lymph nodes may result in FOXP3^+^ lymphocytes accumulation through migration, expansion, or peripheral conversion. As shown in Figures 2A and 2B, FOXP3^+^ lymphocytes mainly accumulated in the periphery of metastatic foci. FOXP3^+^ lymphocytes accumulation in the lymph nodes may promote tumor cell infiltration into the systemic circulation and accelerate the progression of pancreatic cancer through inhibition of effector T cell function.

The prognostic value of measuring FOXP3^+^ T cells in different gastrointestinal cancers remains controversial. In a recent published meta-analysis, it was shown that tumor-infiltrating FOXP3^+^ T cells correlated with poor prognosis in hepatocellular carcinoma and gastric cancer, but good prognosis in colorectal cancer [21]. In the setting of pancreatic cancer, it was demonstrated that tumor-infiltrating FOXP3^+^ cell serves as negative prognostic factor [10], [12], [22]. In our study, univariate analysis demonstrated that the lymph node status and local tumor FOXP3^+^ lymphocytes density were prognostic factors of pancreatic cancer in patients after radical resection. No difference in the prognosis stratified by pathological stage was seen in the 50 pancreatic cancer patients. After factoring in the lymph node status and local tumor density of FOXP3^+^ lymphocytes, multivariate analysis indicated the latter as having only borderline statistical significance. In this patient set, the local tumor FOXP3^+^ lymphocytes density appeared to have greater prognostic value than even nodal status.

In conclusion, we confirmed that the accumulation of FOXP3^+^ lymphocytes in the primary tumor tissue correlates with lymph node metastasis in pancreatic cancer. Importantly, we also demonstrated that the presence of FOXP3^+^ lymphocytes in regional lymph nodes is associated with nodal metastases and that metastatic lymph nodes contain significantly more FOXP3^+^ cells than do negative lymph nodes.
